# Plasmodial slime moulds (myxomycetes) in Swedish and Nordic folk biology

**DOI:** 10.1186/s13002-024-00740-6

**Published:** 2024-11-12

**Authors:** Ingvar Svanberg, Sabira Ståhlberg

**Affiliations:** Institute for Russian and Eurasian Studies, Uppsala University, Box 514, 751 20 Uppsala, Sweden

**Keywords:** Cultural ecosystem services, Ethnomycology, Folk beliefs, Historical ethnobiology, Local knowledge

## Abstract

**Background:**

Folk biology commonly contains knowledge of many more taxa than those of immediate economic importance. Species with little or no practical use are, however, often overlooked by ethnobiological research. An example are a few Myxomycetes taxa which played an important role in the folk biology and beliefs of pre-industrial Sweden and adjacent Nordic countries, Denmark, Norway and Finland. Such organisms are not of less interest for the understanding of human-biota relationships; local knowledge about the entire biota in a given environment should therefore be studied to comprehend the full range of folk biology.

**Methodology:**

This qualitative study analyses and reviews historical data available in archives and published ethnographic collections as well as scattered and fragmentary notes in the literature using a historical ethnobiological approach.

**Results:**

Peasants in the Nordic countries recognized three taxa of myxomycetes. Scrambled egg slime, *Fuligo septica* (L.) F.H.Wigg., in particular attracted interest and is known by many local folk names. This slime had no practical value or use, yet it was well known in folk biology and associated with a supernatural malevolent being which in the shape of a hare or cat stole milk or butter on behalf of a witch. The belief in evil origins of slime became the cause of violent actions such as whipping and burning of the organism. Two other taxa, *Lycogala epidendron* (L.) Fr. and *Mucilago crustacea* F.H.Wigg., have also been observed in folk biology, but data on how they were perceived and treated is sparse.

**Conclusions:**

The sudden appearance of strange shapes and clear colours of myxomycetes in damp weather created both fear and curiosity; these odd organisms required explanations, interpretations and actions. Our example of the economically irrelevant myxomycetes in Sweden and nearby Nordic countries indicates that interpretations in pre-industrial societies of natural phenomena and various organisms, connections with beliefs and perceptions about the surrounding environment and the possible consequent actions should be studied alongside economic plants and animals in ethnobiological research, for a deeper understanding about folk biology and the multilayered and multidimensional relationships between humans and biota.

## Introduction

Certain organisms attract human interest without obvious economic benefits. This relationship should be of equal interest to ethnobiologists: ethnobiology is the scientific study of human perception of biota found in the surrounding landscape [1], the interaction between humans and other organisms, and the cultural expressions arising from this interaction. Folk biology may be defined as an understanding of the biological world: how human groups perceive, interpret and reason about living kind [2], and how they categorize, explain and understand biota in their environments at different points in time. Questions of *how*, *when*, *where* and *for whom* an organism has been important are crucial. Not only economically interesting species are of significance to humans; therefore, the entire surrounding biota should be studied [3] and it should be analysed not only in separate segments but also as an interconnected whole.

Why do taxa without any clear economic or practical use attract human attention? A keen interest in and curiosity about the environment can be observed especially in pre-industrial societies who depended on nature to a much higher degree than present-day urbanized and industrialized societies [4]. An important factor for interest is the *unusual*. Emotional associations can explain some ideas about plants: for instance, strong fears, needs or desires can be associated with an existing or occurring external, conspicuous or strange factor or element. Carl-Vilhelm von Sydow notes the importance of trees in the cognitive reality of pre-industrial peasants in Sweden [5]: an odd or otherwise spectacular tree was assigned a role in folk medicine rites (*tandvärksträd ‘*toothache tree’); another tree had a taboo attached to it or ‘permitted’ the passer-by to have an alcoholic drink (*suptall* ‘schnapps pine’, usually a solitary pine tree along the road) [6]. Similarly, small organisms with a peculiar appearance or behaviour could attract attention without providing any immediate benefits [7]. ‘The first’ or ‘last’ in various contexts, such as the first sighting of a certain flower in spring, are common in folk biology [5, 8]. Such first or last plants were often ascribed health-giving or divinatory properties for the forthcoming harvest, expected arrival of winter, etc. [9]. All organisms recognized and named by humans provide various kinds of cultural ecosystem services, non-material benefits humans obtain from ecosystems through spiritual enrichment, cognitive development, reflection, recreation and aesthetic experiences [10], as well as folk and religious beliefs and various understandings, explanations and interpretations about the surroundings.

Research in historical folk biology and folk knowledge provides not only information about economically important organisms, but also about less known past relationships with biota, practices and uses. These ‘invisible’ aspects are as important as the ‘visible’ or obvious and easily identifiable human-biota relations, and therefore should be studied both separately and together with organisms of obvious practical use. One such example is the naming and local knowledge and belief in the origins of myxomycetes, an irrelevant and economically useless organism for humans in pre-industrial Sweden and its neighbouring countries: in the context and with the methods of historical ethnobiological analysis, and by employing a qualitative method in which ethnographic description plays an essential role, we are able to uncover not only a wealth of folk knowledge, interpretations, beliefs and religious connotations, but also several layers of human relations towards biota, including motivations for the destruction of specific organisms [9].

Among the many organisms with multiple and multilayered significances in the folk biology of peasants in Sweden and neighbouring Nordic countries, Denmark, Norway and Finland, are a few plasmodial slime moulds, Myxomycetes (Syn. Myxogastria) [11]. They are indeed weird beings: myxomycetes have some features in common with fungi and others with animals. They are clearly distinguished from other organisms by their morphology and temporality and their brightly coloured slimy bodies, which rapidly change shape and size as they grow. They do not resemble animals, plants or fungi, yet they are easy to spot when they suddenly appear on decaying wood in the vicinity of human settlements. A slime mould can move across substrates like a large amoeba while consuming everything in its path, including bacteria, spores and other organic materials [12]. About 400 taxa, distributed over 57 genera, have been documented in these Nordic countries [13, 14]. SLU, the Swedish Species Information Centre (*Artdatabanken,* Species data bank), reports 239 taxa for Sweden [15]. Myxomycetes are usually found in dark and damp environments in the forest, where they grow on rotting trees and branches, bark, moss and decaying leaves. They can be observed either in the motile stage, so-called plasmodium, or as fruiting bodies. The slime coat protects the plasmodium from drying out and thus, when crawling, myxomycetes usually leave a trace of slime [16].

Scientific interest in these organisms dates back to the mid-seventeenth century: the German physician Thomas Panckow presented wolf’s milk in *Herbarium Portatile* in 1654; the first scientific description of *Fuligo septica* was given by the French botanist Jean Marchant in 1727, who called it *fleur de tan* ‘bark flower’ and classified it as a sponge [17]. Carl Linnaeus named the same taxon *Mucor septicus* in *Species Plantarum* (1763) [18]. The famous Swedish mycologist Elias Fries described numerous slime moulds as Myxogastres in 1829 [19].

The peasants in the Nordic countries commonly recognized three species among Myxomycetes: scrambled egg slime, *Fuligo septica* (L.) F.H.Wigg.*,* wolf’s milk, *Lycogala epidendron,* (L.) Fr., and dog sick slime mould**,** *Mucilago crustacea* F.H.Wigg. All three were associated with folk beliefs until the late nineteenth century [20]. The peasants had no use for the slime moulds, yet these organisms played an important role in folk beliefs about witches and magic. In folk biology, slime moulds were connected with milk and butter, both important ingredients in the peasant diet; a witch who specially created a supernatural being for stealing these crucial ingredients; and the action of vomiting or spilling the stolen goods by the magical hare or cat while it was bringing the food back to the witch. Belief in the evil origins, with the devil at the root of it all (witches were supposed to contact directly with him), caused the peasants to destroy the organisms in various ways.

**Fig. 1:**
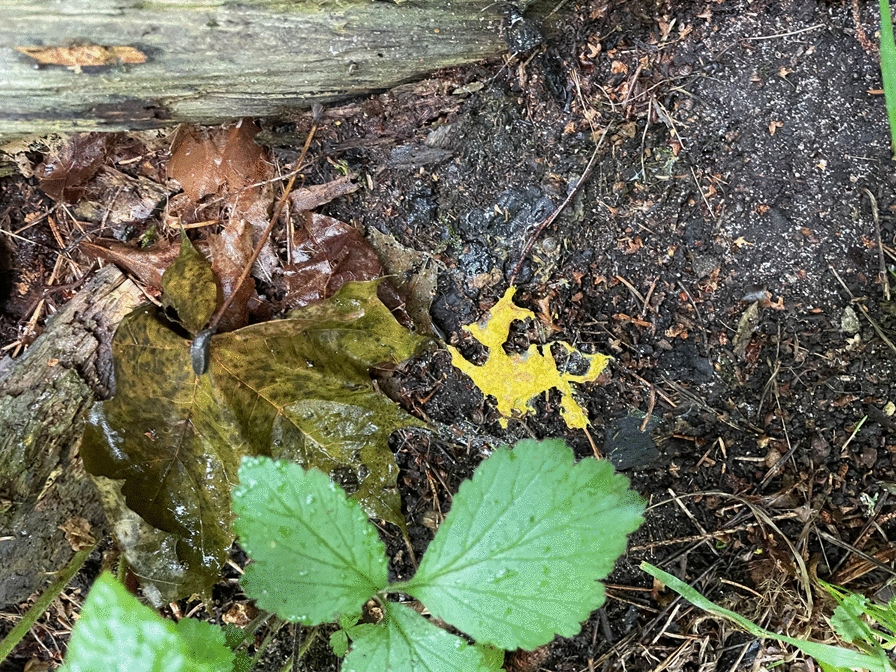
*Fuligo septica* can appear in different shapes, sizes and yellow nuances. Häverödal, Uppland province, central Sweden (Photo © Ingvar Svanberg, 17 July 2024)

The earliest sources on *trulsmör* ‘troll/witch butter’ (*Fuligo septica*) in Sweden date to the sixteenth century; one source from September 1597 connects it with a witchcraft case [21]. (Fig. 1) A Norwegian source mentions *trollkattsspyor* ‘troll/witch-cat puke’ in 1658 [22]. *Trulsmör* is still known colloquially as *trollsmör*. In Swedish (including Finland-Swedish and Estonia-Swedish) and other Scandinavian languages (Danish, Norwegian), *troll* signifies both ‘troll’, a hairy, small-size forest being with a tail and limited magic capabilities, and ‘witch’, human, usually a woman. The verb *trolla* means ‘to bewitch, to use magic’. Other folk names of slime moulds are related to carrying, because the organisms were perceived as traces (vomit) of the supernatural being carrying stolen milk: *bäreskarn*’carrier filth’ is mentioned in a source from 1617, *bjärsmör*’carrier butter’ in a lexicon from 1630 and *pukelort* ‘small-devil dirt’ from 1672 [23]. The Swedish verb *bära* means ‘to carry’; *bära, bjära* for ‘[milk-hare/cat] carrier’ has in Finnish and the northern Meänkieli (Tornedalian Finnish) transformed to *para*. Globally, definitions of slime moulds as products of evil forces are very old: in a Chinese-language source from the fourth century, a substance that could be *Fuligo septica* was called *gui shi* ‘demon faeces’ [24].

This study discusses the folk biology related to slime moulds, a specific human-biota relationship, and the effects of folk beliefs on the organisms. Few ethnobiologists have taken more than a cursory interest in these organisms: Vagn J. Brøndegaard 1979 and N. Floro Andrés-Rodríguez & Oscar Requejo 2020 provide a broader comparative perspective from several countries [25, 26]. A brief review of Swedish data is presented by Ingvar Svanberg in 2005 [27]. María Mercedes Rodríguez-Palma et al. published in 2017 an interesting paper on myxomycetes used as food in Mexico, a practice unknown in the Nordic countries [28].

## Materials and Methods

This study is primarily based on pre-twentieth-century data available in dialect and folklore archives and published ethnographic collections in the Nordic countries. Data from archives and scattered notes in literature are invaluable sources for mapping out the diversity of pre-industrial human relationships with their surrounding biota. This process of mapping becomes more detailed for the fairly well-documented eighteenth and nineteenth centuries; for earlier periods sources are scarce and fragmented, and often less reliable [9]. Several kinds of sources have been used: ethnological monographs, folk-life records, mycological literature, philological studies and topographic literature. With the help of ethnographic records in printed sources, dictionaries and oral records in folklore archives in Sweden (Lund and Uppsala) [7, 27], this article is an attempt to review and analyse the cultural context, knowledge and use of various myxomycetes [29].

## Results

### Scrambled egg slime, *Fuligo* septica (L.) F.H. Wigg (1780)

This is the most well-known myxomycetes in the Nordic countries. This yellowish organism appears suddenly on tree trunks and other decaying substances in wet or damp weather, and it can grow up to twenty centimetres wide and three centimetres thick [30]. This slime mould has a worldwide distribution [31]. It is a very conspicuous and colourful organism: the aethalia or fruiting bodies are known under many local names in Scandinavian and Finno-Ugric languages and often vary according to province **(**Table 1**).**

**Table 1:** Vernacular names for *Fuligo septica* *Sources*: [25, 27, 34, 36, 44, 48]

*Swedish*
Baradret ‘carrier shit’ (Dalarna)
Baraskit ‘carrier shit’ (Dalarna, Medelpad, Västerbotten, Finland-Swedish)
Bjäradynga ‘carrier-hare dung’ (Jämtland, Ångermanland, Västerbotten, Lappland, Finland-Swedish)
Bjärsmör ‘carrier butter’ (Småland, Östergötland, Gotland, Öland)
Bjäraspy ‘carrier vomit’(Östergötland, Småland, Finland-Swedish)
Bärudrit ‘carrier filth’ (Dalarna, Öland)
Hexspott ‘witch spit’ (Estonia-Swedish)
Käringasmör ‘old hag butter’ (Närke)
Mardret ‘mare filth’(Dalarna, Finland-Swedish)
Mjölkharaspy ‘milk-hare puke’ (Östergötland)
Pukdynga ‘small-devil dung’ (Dalarna, Värmland. Västmanland, Härjedalen, Jämtland, Medelpad, Estonia-Swedish)
Puk-lort ‘small-devil dirt’ (Jämtland, Härjedalen)
Puk-rusa ‘small-devil dropping’ (Härjedalen)
Pukskit ‘small-devil shit’ (Jämtland, Härjedalen, Medelpad)
Puksmör ‘small-devil butter’ (Gotland, Värmland)
Skrattskit ‘devil shit’ (Estonia-Swedish)
Trolldret ‘troll shit’ (Estonia-Swedish, Dalarna, Småland)
Trollkattskit ‘troll/witch cat shit’ (Jämtland)
Trollkäringdret ‘witch hag filth’ (Dalarna)
Trollkäringsmör ‘troll/witch hag butter’ (Uppland, Finland-Swedish)
Trollsmör ‘troll/witch butter’ (Skåne, Blekinge, Halland, Småland, Bohuslän, Västergötland, Södermanland, Närke, Västmanland, Gästrikland, Norrbotten, Åland Islands, Finland-Swedish)
Trollspy ‘troll/witch puke’ (Östergötland, Estonia-Swedish)
Trulskid ‘troll/witch shit’ (Estonia-Swedish)
Tusslort ‘troll dirt’ (Dalarna)
*Norwegian*
Trolkattsmør ‘troll/witch-cat butter’
Trollkattspy ‘troll/witch cat puke’
Troll-lort ‘troll dirt’
Trollkjerringsmør ‘troll/witch-hag butter’
Trollkjerringspot ‘troll/witch-hag spit’
Trollkjerringsspy ‘troll/witch-hag puke’
Trollsmør ‘troll/witch butter’
Tusselort ‘troll dirt’
*Danish*
Heksefedt ‘witch fat’
Heksesmør ‘witch butter’
Troldsmør ‘troll/witch butter’
Troldspyt ‘troll/witch spit’
*Finnish*
Paran oksennus ‘carrier vomit’
Paranpaska ‘carrier shit’
Paranvoi ‘carrier butter’
*Meänkieli*
Paranpaska ‘carrier shit’ (Norrbotten)
*North Sami*
Smiergáhtu baiku’butter-cat shit’ (Sápmi)

In Nordic pre-industrial societies before the end of the nineteenth century, if milk suddenly became scarce, peasants suspected that someone used magic to steal the milk or their ‘butter luck’. In 1845, the Swedish lawyer and antiquarian Richard Dybeck wrote:

“Common is the probably ancient belief that the mould [scrambled egg slime] is left by the Trolls [witches]. It is believed that when it is damaged, the troll [witch] also suffers. Therefore it is not uncommon in remote villages to throw *trollsmör* [‘troll butter’] on the fire and burn it, and to beat or whip it with soft sticks around the flame. It is believed that this will destroy the witch (*trollpackan*), and through the flogging one can torment her even more.”[32].

The mere sight of the yellow substance with its strange shape and bright colour probably created both fear and horror besides curiosity among the peasants. It was widely believed throughout Sweden that this myxomycetes, suddenly appearing on stumps and old house walls, was the thrown-up milk from the stomach of *bjäran*, *mjölkharen* or *puken*, a supernatural creature which was believed to do great harm to livestock. This being had been created by an evil female, that is a witch in the neighbourhood. A certain sign that a *bjära* had visited a cattle barn or sheepfold was the presence of its ‘dung’ or myxomycetes in or near the building [27].

**Fig. 2:**
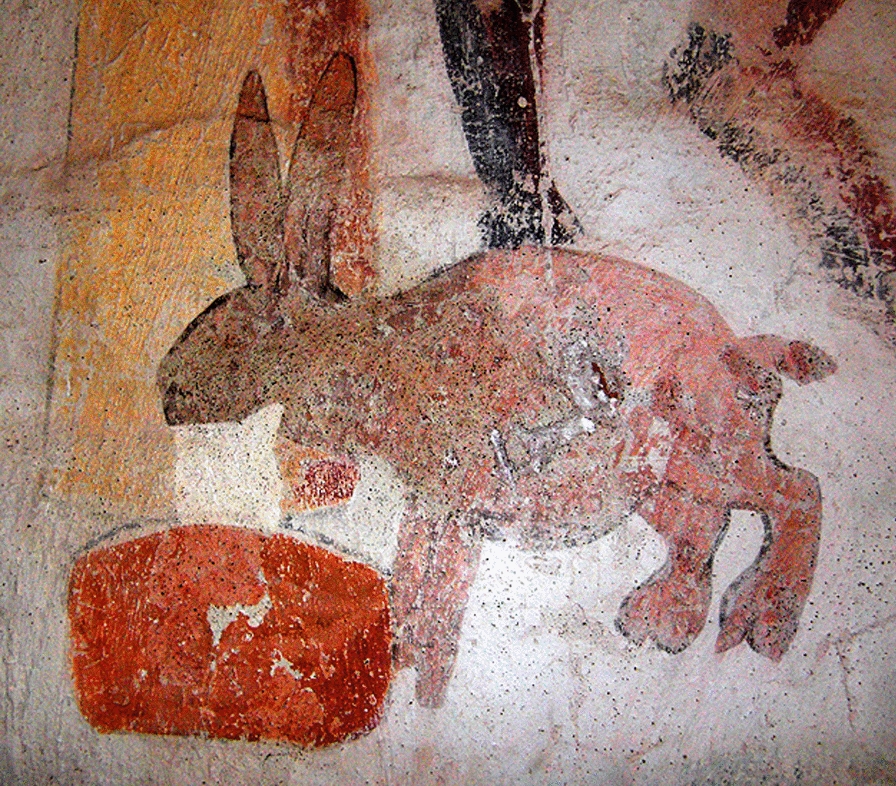
A milk-hare throwing up milk into a receptacle. The milk-hare in Swedish folk belief was a magical creature who stole milk from cows. Late fifteenth-century painting by Albertus Pictor in the porch of Härkeberga Church, Uppland, central Sweden. (Photo Gunnar Creutz, Creative Commons License CC-BY-SA-3.0, Wikimedia)

This milk- and butter-stealing *bjära* or *mjölkhare* ‘milk-hare’ was associated with milking and availability of milk and butter. When cows for some reason yielded less milk, a supernatural thief was blamed: someone poached on the cattle and stole milk and/or butter. These sinister doings were often pinned on a neighbour, commonly a woman. She had a hare, cat, bird or other animal or even a ball of yarn with magical powers in her service. This witch would create her servants out of various materials, including butter, ashes, soil, blood from her own fingers, etc., and she was in league with the devil himself. The earliest evidence of *bjära* beliefs is found in medieval church paintings, most famously from Ösmo church in Södermanland (now Nynäshamn municipality), dating to the 1450 s, and Härkeberga church in Uppland from the late fifteenth century. They show a hare throwing up milk into a basin while the devil is looking on (Fig. 2). The traces of the creature could be identified from the yellowish slime mould growing out of the ground after rainfall: the mass or lumps suddenly appearing on walls, floors, wooden vessels in or around the farm were sure signs that the creature had gorged too much milk and threw it up on stumps and stones when returning home to the witch. Such evil had to be dealt with directly so that the cows could provide milk again for the family. Medieval church paintings also depict milk-handling women consorting with the devil, and butter is the woman’s main tribute to him. By destroying the slime left by her animal servant, actually a part of her as it contained also her blood, she had to appear herself from hiding and the magic was destroyed [33, 34].

Records of burning slime moulds are found in court records starting from the latter part of the seventeenth century. Burning is mentioned in more than half of the extensive materials on the subject. From Södermanland, a nineteenth-century source stated that rods for whipping were made from nine types of broad-leaf trees. Other records mention seven varieties; both are magical numbers. At the end of the eighteenth century, the teacher Johan J. Törner explained that one should whip slime moulds with hawthorn branches or boil the troll butter in an earthenware vessel [35]. In the northern province of Jämtland, the slime moulds must be burnt at a crossroads, while in Värmland they were locked up ‘behind three thresholds and three locks’ so that they would reveal the malevolent person making magic [36].

A detailed description of how to deal with slime moulds is provided by the priest Johan Peter Wallensteen in a description of the parishes of Lidingö and Danderyd around Stockholm at the end of the eighteenth century:

“Newly grown fungi on old fences or walls are called *trollsmör* [‘troll/witch butter’]. It is believed that they are created by an evil person who wants to destroy the barn or churned milk. These fungi are whipped and beaten, as if in anger, while swearing and cursing the wicked man [the devil], and finally with much enthusiasm and hurriedly, the people prepare a fire in the fireplace, throw into it the rods and branches they have used to whip the fungi, scrape carefully with sticks whatever is left in the first place [where they whipped the slime mould]; they are disgusted to touch [it] with bare hands or clothes, and have fun watching how these fungi boil and burn in the fire, and they are very happy about having discovered the evil and about the victory they have gained over it” [37].

Folk names reflect the view that slime moulds were droppings, butter or vomit of a supernatural witch hare or cat milking cows and filling itself up with the milk. In the north of Sweden and Swedish-speaking Österbotten (Ostrobothnia) in Finland, they were called ‘shit’, ‘turd’, ‘dirt’, etc., while in the central provinces of Götaland and Svealand and the southern Swedish-speaking areas in Finland they were called ‘butter’ [34, 38, 39]. In Jämtland, northern Sweden, *pukdynga* ‘small-devil dung’ showed with certainty that the creature had visited the cattle shed [34, 40]. In Ångermanland, it was called *bäradynje* ‘*bjära* dung’ and showed that a *bära* or *bjära* had its home nearby and could therefore reveal its owner [41]. In Edsberg in the province of Närke, the priest Daniel Harbe explained that ‘witch butter’ is a yellowish organism growing on stumps; it was not to be touched, as a witch had dropped butter s/he had conjured up [42]. In all other regions, witches used the help of a *bjära,* whose butter or faeces were the yellow ‘droppings’ in nature [34, 43].

Reports on practical uses of myxomycetes in Sweden are non-existent; their utilization was all connected with magic. Törner recorded in the eighteenth century that if one spread *trollsmör* ‘troll butter’ on the back of a ship, one could have any weather one wished [36]. It could also be used for reversing magic: by carrying myxomycetes into another person’s property, this property in turn was affected by the evil spell, as reported from nineteenth-century Södermanland [44]; there are several examples of magical use from Norway and Denmark as well [25]. Finnish peasants thought it was poisonous to livestock and connected with sorcery: to counteract this, they boiled myxomycetes in tar, salt and sulphur [45]. In Bohemia, central Europe, *Hexenbutter* (German ‘witch butter’) was used as a lubricant for wagon wheels, ‘for as long as any of it lies in the wheel hub, the witch feels the pain’ [46].

### Wolf’s milk, *Lycogala epidendrum* (L.) Fr.

**Fig. 3:**
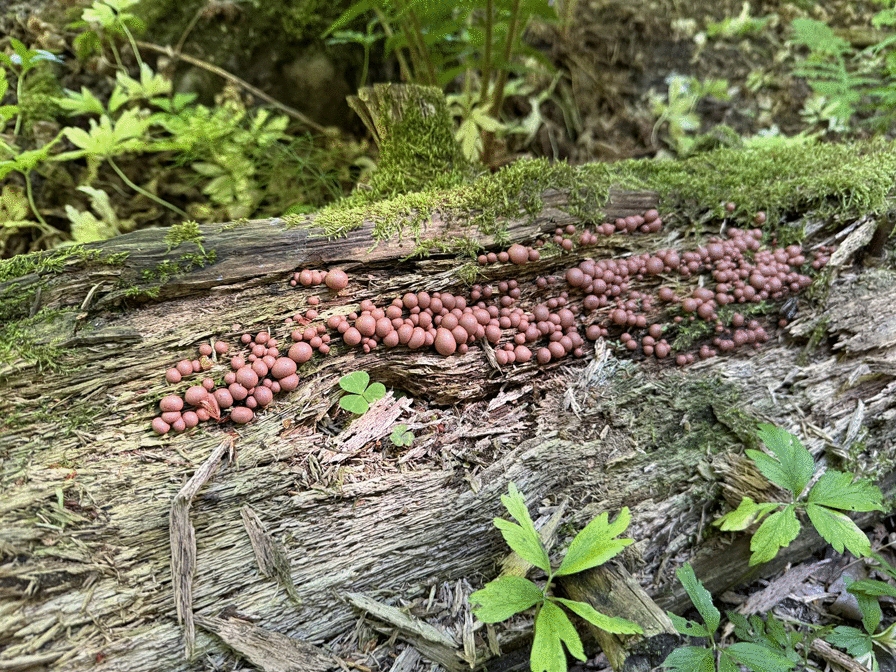
Wolf’s milk, *Lycogala epidendrum.* Billingen, Skövde, Västergötland province, central-southern Sweden. Wolf’s milk is usually red in colour. (Photo © Björn Bråvander, 4 June 2024)

Another well-known slime mould species is wolf’s milk. **(**Fig. 3**)** It forms small, pink to brown-grey fruit bodies some three to fifteen millimetres in size. The intensely red plasmodia can sometimes be observed as a moving layer. Wolf’s milk is common in most of Sweden from spring to autumn. The name was first recorded in 1816 [47]. Similarly to scrambled egg slime mould, the folk names relate to witches; the modern name *vargmjölk* translates as ‘wolf’s milk’ and relates to wolves which are dangerous animals, but it is not related to sorcery. In Norway it was known as *trollkjerringsgrøt* ‘troll/witch porridge’, *trollkjerringsmør* ‘troll/witch butter’ and *rødert* ‘red pea’ [48]*.* Folk taxonomy in Sweden appears not to have distinguished clearly between *Fulica septens* and *Lycogala epidendron,* but some records state that the lumps of the latter were reddish and similar to blood if squeezed. A source from Småland states that witch butter turned to blood if it was cut with a knife forged from nine types of steel [49, 50]. Similar traditions about cutting slime moulds can be found in other Nordic countries, too [48].

### Dog sick slime mould (*Mucilago crustacea *P.Micheli ex F.H.Wigg., 1780, Syn. *Mucilago* spongiosa)

**Fig. 4:**
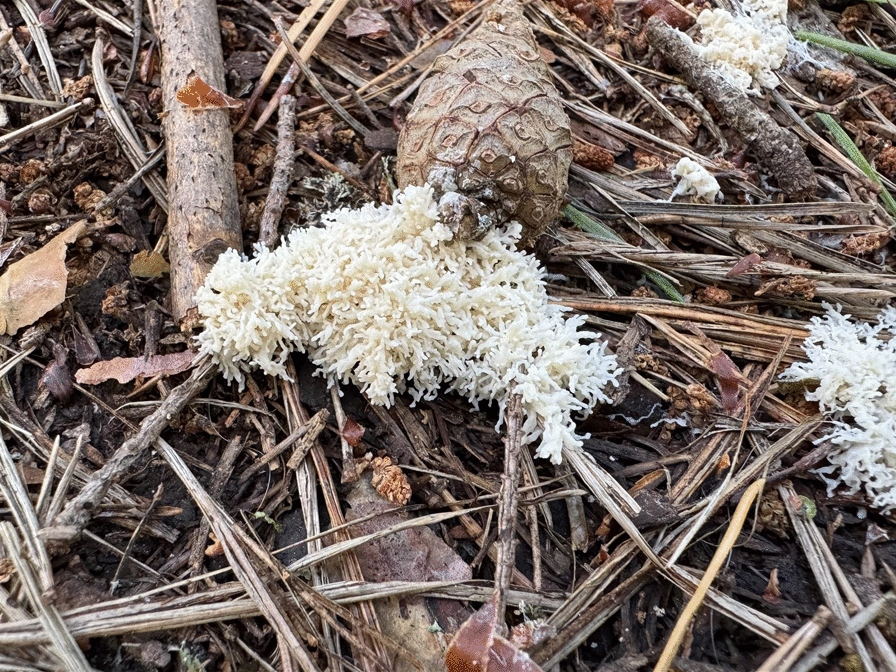
Dog sick slime mould, *Mucilago crustacea.* Danmark parish, Uppland province, central Sweden. (Photo © Björn Bråvander, 9 July 2021)

This slime mould is known as *jordspott* ‘soil spit’ in Swedish and *paransylki* ‘carrier’s spit’ in Finnish. It forms white or yellowish-white plasmodia a few centimetres in length. **(**Fig. 4**)** It is found on grasses, mosses and decaying wood. There are few ethnographic records, but in Edsele parish in the province of Ångermanland it was called *trollspott* ‘troll spit’. Both names are recorded already in 1816 [47]. In the report from Edsele it was not clear whose spit it was: ‘When old people were out walking and saw troll spit in the grass, they said “Yes, last night they have been out, indeed’. I never heard anyone say that the trolls were out, it was only “they”’ [51]. In Denmark it was known as *trollkattsspy* ‘troll/witch cat vomit’, yet if it ended up in cattle feed, it brought good luck to the livestock [48].

## Discussion

While *Fuligo septica* is well covered in historical and archival records and ethnographical studies, the data on *Lycogala epidendrum* and *Mucilago crustacea* are more scarce in the sources. Today, it is almost impossible to know whether this is due to an oversight on the part of researchers and documenters, who focused mainly on economic uses of organisms, or if the two latter ones received less attention from the Nordic peasantry than the conspicuous yellow slime mould; a comparison shows that peasants did not pay the same attention to all myxomycetes in the same way. Primarily vernacular names are documented in ethnographic, historical and mycological source materials. In the case of *Fuligo septica*, there are many names not only in Swedish, including Finland- and Estonia-Swedish, but also in closely related Danish and Norwegian, as well as in the Finno-Ugric Finnish, Meänkieli (Tornedalian Finnish) and Sámi. Similar folk names are known in German, French, English and other European languages, too [25, 34]. The vernacular names in the Nordic countries and at least the German-speaking areas indicate that slime moulds were commonly seen as traces of a magical hare or cat. For *Lycogala epidendrum* and *Mucilago crustacea* records are considerably fewer, but the fact that they also have similar names, connotations and folk beliefs in the Nordic countries shows that they have attracted human interest, although far less than the bright yellow slime mould.

**Fig. 5:**
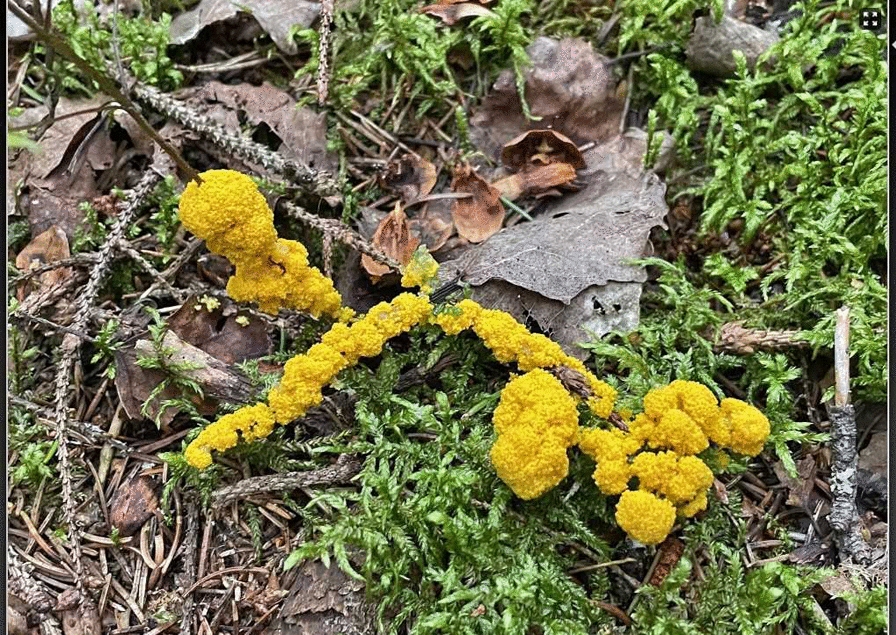
The yellow *Fuligo septica* is known by many local folk names in northern Europe. It had no practical use for humans, but was nevertheless well-known in folk biology: it was seen as the tracks of a supernatural creature, a hare or cat, who stole milk or butter on behalf of a witch and vomited it while running to the witch’s abode. Edvik, Norrala, Hälsingland province (Photo © Alf Pallin, 21 August 2022)

The sudden appearance, strong colour and behaviour (movements) of slime moulds must be explained in some way. The appearance of the yellow *Fuligo septica* was associated with butter and with a creature believed to stealthily milk the cows in the barn during the night, so that the cows ‘dried up’ the next day **(**Fig. 5**)**. As the *bjära* was believed to suck milk from cows or to steal butter and carry the food in its stomach to its owner and creator, regurgitating part of it on the way in the form of this yellow smear, the similarity in the physical shape and colour of *Fuligo septica* (melted butter), and the association with milk and churned milk in the form of butter may explain why this myxomycete was more popular in folk biology than the other two taxa.

In folk biology, the slime moulds have been associated with the supernatural, a belief in witches, magical creatures, sorcery and spells mixed with Christian religion (the devil). The beliefs were kept alive among the peasants for several centuries and almost into modern times. In Sweden, sorcery was not a serious crime (except when someone was proven to have been murdered by magic) until the Reformation at the beginning of the sixteenth century. Only after the mid-1600 s witch-hunts became more common in Sweden, but in comparison with several Central and South European countries, witch purges were few in the Nordic religious-cultural sphere. They were often influenced and inspired by stories and beliefs from abroad, brought by merchants and students from Sweden-Finland (a single country between the twelfth century and 1809), who studied in Central Europe and especially in Germany after the Reformation [23, 52]. Folk beliefs about witches and their supernatural servants existed throughout Sweden; the tradition that slime mould was a trace of a thieving creature survived longest in upper Norrland in the far north [34, 53].

The slime was helpful, actually, before being destroyed as a means to torment and finding out the witch: one could reveal who in the neighbourhood was keeping a *bjära* by following the track of yellow stains*.* Often she was identified as a female who had direct dealings with the devil and the sorcery skills necessary to create a carrier animal. The witch woman’s gift and payment for access to the devil was butter, a product which at least since the thirteenth century was used also as tax payment in Sweden. Butter was an ingredient in the creation process of her animal servant, in addition to various other materials including soil, metals and her own blood. Butter has been appreciated for centuries; it was and still is valuable in North Europe, and there are many popular sayings and proverbs in Swedish about it [54].

Links between *Fuligo septica* and milk-stealing evil creatures and identification as milk or vomit exist also elsewhere than in the Nordic countries and Estonia in north-western Europe, including Latvia and Germany, and among various communities in North America. Ethnographic and mycological literature from outside this region however yield meagre results, either because documenters and researchers were not interested or because valuable information has not been spotted in the available literature [55, 56]. Possibly the specific north and north-western European traditions are regional; this must be verified through a global comparison.

There is however reason to encourage interest in myxomycetes and other economically irrelevant species in connection with ethnobiological fieldwork or when searching for data in archives and literature. As slime moulds are of no practical interest, researchers often overlook them and documenters have ignored them as not useful. Human relationships with organisms may look insignificant and not be caught in the overly coarse mesh researchers often use for data collection. Once one starts asking questions about certain species, intriguing aspects of cultural history can be uncovered [7, 57]. Yet, in the absence of documentation, many interesting aspects are lost and especially about the relationship between humans and biota.

## Conclusion

Though it is not obvious for a general public today, and it was certainly not clear to pre-industrial peasants, curious about plasmodial slime moulds and wishing to explain them, myxomycetes provide various ecosystem services yet to be fully understood, and they also offer cultural ecosystem services. In the cognitive reality of Nordic peasants, they were a sign of odd happenings and associated with beliefs and religious elements. Although it is not on record in ethnographic archives and publications, peasants must have observed and known that where slime moulds appeared, there was increased humidity and decaying matter such as wood or leaves. The sudden appearance and colour of the *Fuligo septica* still arouses great fascination especially among children.

A comparative analysis with Central and South European beliefs and accounts of slime moulds could be fruitful for the understanding and conceptualizing the role of myxomycetes in pre-industrial societies. The example of how humans related to myxomycetes in Sweden and neighbouring Nordic countries, including the interpretation of the unexpected appearance, connection of the organisms with the failure of an important food resource and economic activity (milking), interpretation of the situation as related to magical beliefs and taking action (whipping, burning) to destroy the perceived evil, show that these economically insignificant organisms were laden with rich cognitive meanings which captured and triggered human imagination. The slime mould example also indicates that interpretations in pre-industrial societies of natural phenomena and various organisms, their connections with beliefs and perceptions about the surrounding environment and possible consequent actions should be studied alongside economically useful and utilitarian plants and animals. A broader study of biota and their significance for humans, obvious or not so overt, provides a better understanding for the ethnobiological study of folk biology and highlights not only the multilayered but also the multidimensional relationships between humans and their surrounding biota both in history and at present.

## Data Availability

The authors confirm that the data supporting the findings of this study are available within the article. No data sets were generated or analysed during the current study.
